# Impact and Determinants of Structural Barriers on Physical Activity in People with Cancer

**DOI:** 10.1007/s12529-021-10014-0

**Published:** 2021-09-22

**Authors:** Johanna Depenbusch, Joachim Wiskemann, Alexander Haussmann, Angeliki Tsiouris, Laura Schmidt, Nadine Ungar, Monika Sieverding, Karen Steindorf

**Affiliations:** 1 Division of Physical Activity, Prevention and Cancer, National Center for Tumor Diseases (NCT) Heidelberg and German Cancer Research Center (DKFZ), Im Neuenheimer Feld 581, 69120 Heidelberg, Germany; 2 Medical Faculty, Heidelberg University, Im Neuenheimer Feld 672, 69120 Heidelberg, Germany; 3 Division of Medical Oncology, National Center for Tumor Diseases (NCT) Heidelberg and University Clinic Heidelberg, Im Neuenheimer Feld 460, 69120 Heidelberg, Germany; 4 Department of Psychosomatic Medicine and Psychotherapy, University Medical Center Mainz, Johannes Gutenberg University Maiz, Untere Zahlbacher Straße 8, 55131 Mainz, Germany; 5 Institute of Psychology, Heidelberg University, Hauptstraße 47-51, 69117 Heidelberg, Germany

**Keywords:** Cancer, Exercise oncology, Impediment, Physical activity, Structural barriers

## Abstract

**Background:**

A better understanding of the role of structural barriers for physical activity (PA) after a cancer diagnosis could help to increase PA among people with cancer. Thus, the present study aimed to identify determinants of structural barriers to PA in people with cancer and investigate the association between structural barriers and insufficient post-diagnosis PA, taking different PA change patterns into account.

**Methods:**

A total of 1299 people with breast, prostate, or colorectal cancer completed a questionnaire assessing their socio-demographic and medical characteristics, pre- and post-diagnosis PA, and perceived PA impediment by seven structural barriers. Regression analyses were used to investigate determinants of the perception of structural barriers and to examine the association between structural barriers and insufficient post-diagnosis PA, also with regard to different pre-diagnosis PA levels.

**Results:**

Overall 30–60% of participants indicated to feel impeded by structural barriers. The analyses revealed a younger age, higher BMI, lower educational level, no current work activity, co-morbidities, and lacking physicians’ exercise counseling as significant determinants of the perception of structural barriers. Individuals reporting stronger impediments by structural barriers were significantly less likely to be meeting PA guidelines post-diagnosis, particularly those with sufficient pre-diagnosis PA levels.

**Conclusions:**

The study highlights the need for tailored PA programs for people with cancer as well as for more guidance and support in overcoming structural barriers to improve PA behavior.

The study has been registered under NCT02678832 at clinicaltrials.gov on February 10^th^ 2016.

## Introduction

Physical activity (PA) is considered as one of the most effective self-management strategies for people with cancer [1]. As numerous studies have documented positive effects on treatment-related side effects like reduced fatigue and increased quality of life [2, 3] and further indicated an association with decreased risks of cancer recurrence and cancer-specific mortality [4–6], PA is playing an increasingly important role during and after cancer treatment. Accordingly, current guidelines recommend that people with cancer should perform at least 30 min of moderate-intensity aerobic activity three times per week and 20 to 30 min of resistance exercises twice per week [7].

Although the awareness for PA seems to increase and recent studies have reported encouragingly high numbers of sufficiently active people with cancer [8], there is still a considerably large number of individuals who remain insufficiently active or decrease their PA after the diagnosis [9, 10]. To successfully develop and implement interventions aiming to improve PA behavior among people with cancer, it appears crucial to identify barriers that prevent them from performing PA. In this context, Hefferon et al. defined psychological barriers like lack of motivation and fear, physical and disease-related barriers like treatment-related side effects and co-morbidities, and structural barriers like missing access to exercise facilities as the three main themes for not engaging in PA [11]. While previous research has mainly focused on physical and disease-related barriers and identified treatment-related side effects, fatigue, pain or other health-related problems as the most frequently reported barriers to PA across different cancer types [12–15], the role of structural barriers has only scarcely been investigated so far.

Of note, a few studies have suggested that structural barriers might be less prevalent than other types of barriers but nevertheless affect PA behavior most strongly. One study investigating personal, social, and structural barriers indicated that although personal barriers were reported more frequently, structural barriers, i.e., a missing exercise partner and lack of place to perform PA as well as missing access to a gym or equipment and insecurity about what to do, were significantly associated with fewer PA minutes among people with breast and prostate cancer [16]. Results of a study among colorectal cancer patients revealed that structural barriers had the strongest effect on whether individuals met PA guidelines post-diagnosis although they were the least salient barriers [17].

However, these studies have mainly looked at environmental factors, whereas a systematic assessment of different kinds of structural barriers is still lacking. Given the suggested impediment by structural barriers for PA among people with cancer, it appears worthwhile to gain a more differentiated understanding. Aiming to increase PA after a cancer diagnosis by alleviating the perception of structural barriers, it firstly seems important to determine, which individuals perceive structural barriers as particularly impeding for their PA. Some studies have indicated that the perception of overall barriers differs between different cancer types [16, 18] and might be increased among younger individuals with lower educational levels and a higher body mass index (BMI) [15], but potential determinants for structural barriers have been neglected so far. Besides socio-demographic and medical characteristics, it seems likely that physicians’ exercise counseling plays a role for the perception of structural barriers as it could counteract a lack of information and knowledge about suitable exercise programs [19]. Furthermore, the association between different structural barriers and post-diagnosis PA needs to be investigated in more detail. Previous research has shown that individuals who perceive structural barriers are less likely to be meeting PA guidelines after the diagnosis [17]. However, insufficient PA post-diagnosis can result from two different PA change patterns: Becoming insufficiently active, i.e., decrease of PA to insufficient levels among individuals, who were meeting PA guidelines before the diagnosis, vs. remaining insufficiently active, i.e., maintenance of insufficient PA levels among individuals, who were not meeting PA before the diagnosis. Considering this distinction might enable a more specific targeting of PA interventions and therefore help to counteract insufficient PA after a cancer diagnosis.

Thus, the present study aimed to (a) identify to what extent socio-demographic and medical characteristics and physicians’ exercise counseling explained the perception of structural barriers as impeding for PA, (b) investigate whether the perception of structural barriers was associated with insufficient post-diagnosis PA, and (c) explore whether the proposed association between structural barriers and insufficient post-diagnosis PA applied equally to the two possible PA change patterns, i.e. becoming vs. remaining insufficiently active.

## Method

### Design and Participants

The present cross-sectional study was part of the large-scale Momentum project Heidelberg. The survey was conducted between January 2017 and May 2018 and aimed to assess social-cognitive norms regarding physical activity among people with cancer. The study was targeted at individuals who were diagnosed with breast, prostate, or colorectal cancer ≤ 2.5 years ago and who had received cancer treatment, e.g., surgery, chemotherapy, or radiation, or would receive treatment in future. Participants were mainly recruited via the cancer-registry Baden-Württemberg and additionally via physicians, who had participated in our study among health care professionals (HCP), at information events, via self-help group associations and in online portals for people with cancer. Detailed information about inclusion criteria and recruitment strategies are presented elsewhere [8]. The commission of the Faculty of Behavioral and Cultural Studies of the University of Heidelberg granted ethical approval for the study and all participants provided written informed consent.

### Measures

All information were provided by participants as self-report in a paper–pencil or congruent online survey. Socio-demographic and medical variables comprised age, sex, height, and weight for the calculation of the BMI, educational level, current work status, cancer type, date of latest diagnosis, treatment type, and status and a list of co-morbidities. Physicians’ exercise counseling was assessed in accordance to the 5A framework [20]. Participants were asked to indicate which of the five counseling steps (Assess, Advise, Agree, Assist, and Arrange follow-up) their physician had covered. Based on this, a weighted sum score (5A score) was computed which considered the number as well as profoundness of the single counseling steps [21]. For PA assessment, a modified version of the Godin-Shepard Leisure-Time Physical Activity Questionnaire [22] was used, asking how many minutes of light, moderate, and vigorous-intensity PA were performed on average per week before the cancer diagnosis and in the last week. Moderate-to-vigorous PA (MVPA) minutes were calculated as moderate-intensity PA minutes plus two times vigorous-intensity PA minutes.^1^ Participants were classified as not meeting PA guidelines (0–149 min MVPA/week) or meeting PA guidelines (≥ 150 min MVPA/week) pre- and post-diagnosis. Based on this, two change patterns for insufficient post-diagnosis PA were defined: (1) becoming insufficiently active, i.e., meeting PA guidelines pre- but not post-diagnosis, and (2) remaining insufficiently active, i.e., not meeting PA guidelines pre- and post-diagnosis. Items for perception of structural barriers for PA were generated based on our previous qualitative and quantitative study among HCP [33, 40] and pre-tested in a pilot study among 85 people with cancer. Participants were asked to rate to what extent the following 7 factors impeded their PA on a 4-point Likert scale from 0 “not at all” to 3 “very strongly”: (1) lack of information material regarding PA for people with cancer, (2) lack of PA offers specifically for people with cancer, (3) lack of PA offers overall, (4) lack of possibility to clarify if one is medically suitable for PA, (5) lack of a contact person who is specialized in exercise oncology counseling and treatment, (6) lack of therapeutic programs that are reimbursed by health care insurances, and (7) lack of parks, walking, running and cycling paths or public pools in the neighborhood [23]. To compute the number of perceived barriers, the variables were dichotomized as 0 “not at all” vs. 1 “slightly, strongly or very strongly” and values were summed up across all barriers.

### Statistical Analyses

Descriptive statistics were used to determine socio-demographic and medical characteristics of the study population as well as exercise counseling, MVPA, and structural barrier variables. Separate linear regression analyses were performed to identify determinants of the perception of each structural barrier and the number of perceived barriers. To investigate the impact of structural barriers on insufficient post-diagnosis PA, odds ratios (OR) with 95% confidence intervals (CI) were estimated in logistic regression analyses. The seven barriers and the number of barriers were evaluated as interval-scaled predictors independently of each other in separate regression models. The analyses were adjusted for age and sex as well as factors that were found to be significant predictors of post-diagnosis PA in previous studies among the same sample, i.e., BMI, educational level, cancer type, time since diagnosis, co-morbidities, pre-diagnosis MVPA, and 5A score [8, 21]. To further explore differences in the proposed association of structural barriers and insufficient post-diagnosis PA between the two possible change patterns, the sample was split according to whether or not participants were meeting PA guidelines pre-diagnosis. Logistic regression analyses were re-run for each split-sample to determine if structural barriers were associated with (1) becoming insufficiently active among participants who were meeting PA guidelines pre-diagnosis and (2) remaining insufficiently active among participants who were not meeting PA guidelines pre-diagnosis. All statistical analyses were carried out with IBM SPSS version 25. A *p* < 0.05 was considered statistically significant.

## Results

### Descriptive Statistics

**Fig. 1:**
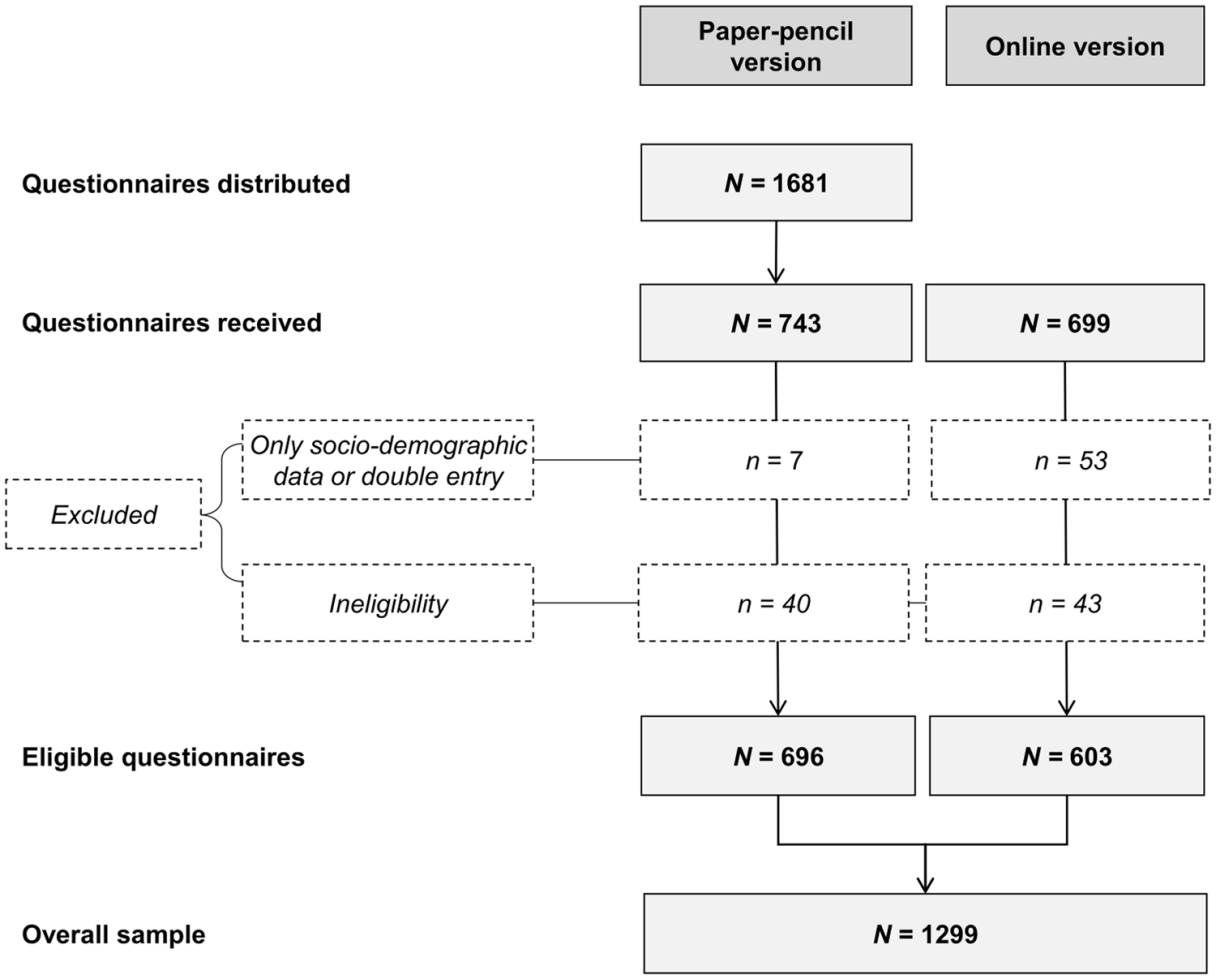
Recruitment flow. Adapted and reprinted by permission from Springer Nature Customer Service Centre GmbH: Springer Nature, Supportive Care in Cancer, Steindorf et al., Change patterns and determinants of physical activity differ between breast, prostate and colorectal cancer patients, © 2019

**Fig. 2:**
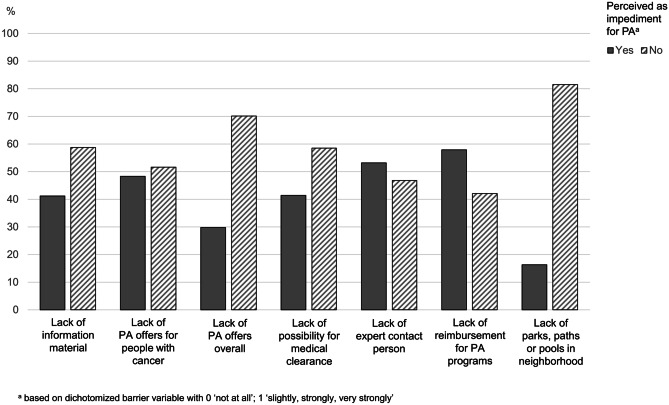
Frequencies of participants perceiving impediment for physical activity (PA) by structural barriers

The recruitment flow of the study is presented in Fig. 1. The overall sample of eligible participants consisted of 1299 people with cancer, of which 631 were diagnosed with breast, 344 with prostate and 324 with colorectal cancer. A total of 754 individuals were female; participants were on average 60.0 years old (*SD* = 12.5) and 14.9 months post-diagnosis (*SD* = 7.6). Further descriptive characteristics of the sample are shown in Table 1. Table 2 displays the perceived impediment for PA by structural barriers. Overall, 30 to 58% of participants indicated to feel at least slightly impeded in their PA; the mean number of reported barriers was 2.8 (*SD* = 2.4). The most frequently reported barrier was “lack of therapeutic programs that are reimbursed by health care insurances” (57.9%), followed by “lack of an expert contact person” (53.2%) and “lack of PA offers specifically for people with cancer” (48.3%) (Fig. 2).

**Table 1:** Descriptive statistics of sample characteristics (*N* = 1299)

	Mean or abs. number	*SD* or %
Age (years)^a^	60.0	12.5
BMI (kg/m^2^)^a^	26.4	4.8
Sex		
Female	754	58.1*%*
Male	543	41.9*%*
Educational level^b^		
Lower	736	57.8*%*
Higher	538	42.2*%*
Current work status^c^		
Not working	844	67.6
Working	405	32.4
Cancer type		
Breast cancer	631	48.6%
Prostate cancer	344	24.9%
Colorectal cancer	324	26.5%
Time since diagnosis [months]^a^	14.9	7.6
Current treatment status		
No treatment	726	57.9%
Receiving treatment	528	42.1%
Surgery		
No	6	0.5%
Yes	1126	99.5%
Chemotherapy		
No	726	57.0%
Yes	548	43.0%
Radiotherapy		
No	559	43.8%
Yes	717	56.2%
Hormone therapy		
No	843	66.6%
Yes	422	33.4%
Co-morbidities		
None	549	44.6%
≥ 1	683	55.4%
Pre-diagnosis MVPA		
0–149 min/week	431	38.0%
≥ 150 min/week	702	62.0%
Post-diagnosis MVPA		
0–149 min/week	522	46.6%
≥ 150 min/week	597	53.4
5A Score for PA counseling^a,d^	1.0	0.9

*SD* standard deviation, *PA* physical activity, *MVPA* moderate-to-vigorous physical activity

^a^Displayed as mean (*M*) and standard deviation (*SD*)

^b^*Lower*: no degree or (lower-) secondary education degree; *Higher*: diploma qualifying for university or university degree

^c^*Not working*: homemaker, retired, on sick-leave or unemployed; *Working*: currently working or student

^d^Weighted sumscore for PA counseling based on 5A framework

**Table 2:** Descriptive statistics of perceived structural barriers

	Not at all		Slightly		Strongly		Very strongly		Mean, SD^a^	
Lack of	*N*	*%*	*N*	*%*	*N*	*%*	*N*	*%*	*M*	*SD*
Information material	679	58.8	308	26.7	113	9.8	54	4.7	1.60	0.85
PA offers for people with cancer	592	51.7	283	24.7	176	15.4	95	8.3	1.80	0.98
PA offers overall	793	70.2	223	19.7	88	7.8	26	2.3	1.42	0.73
Possibility for medical clearance	666	58.6	276	24.3	126	11.1	69	5.3	1.65	0.90
Expert contact person	533	46.8	303	26.6	197	17.3	105	9.2	1.89	1.00
Reimbursement for PA programs	482	42.1	256	22.4	236	20.6	170	14.9	2.08	1.10
Parks, paths or pools in neighborhood	936	81.5	143	12.5	44	3.8	25	2.2	1.27	0.64

Number of barriers^b^	*N*	*%*								
0	347	29.4								
1	109	9.2								
2	106	9.0								
3	121	10.3								
4	145	12.3								
5	139	11.8								
6	134	11.4								
7	79	6.7								

*M* mean, *SD* standard deviation, *PA* physical activity

^a^Based on 4-point Likert Scale with 0 “not at all”; 1 “slightly”; 2 “strongly”; 3 “very strongly”

^b^Based on dichotomized barrier variable with 0 “not at all”; 1 “slightly, strongly, very strongly”. *M* = 2.8*, SD* = 2.4

### Determinants of the Perception of Structural Barriers

Results of the linear regression analyses indicated that a younger age, lower educational level, and no current work activity were significantly associated with stronger impediments by all structural barriers except “lack of parks, paths or pools in the neighborhood” as well as with a higher number of perceived barriers (all *p’s* < 0.05) (Table 3). A higher BMI was revealed as a further significant predictor of five of the barriers and a higher overall number of barriers (all *p’s* < 0.05). With regard to medical factors, people with prostate cancer (*p* = 0.044) and those who had received radiotherapy (*p* = 0.007) perceived a lack of PA programs overall as more impeding for their PA; having received radiotherapy was moreover significantly associated with stronger impediments by a lack of possibility for medical clearance for PA (*p* = 0.005) and a higher number of barriers (*p* = 0.007). Participants with co-morbidities reported stronger impediments by four of the structural barriers and a higher number of barriers (all *p’s* < 0.05). Lastly, a less comprehensive exercise counseling was associated with barriers “lack of information material” (*p* = 0.009), “lack of possibility for medical clearance for PA” (*p* = 0.015), and “lack of expert contact person” (*p* = 0.001).

**Table 3:** Linear regression results on socio-demographic and medical determinants of perceiving structural barriers

	Lack of information material		Lack of PA offers for people with cancer		Lack of PA offers overall		Lack of possibility for medical clearance		Lack of expert contact person		Lack of reimbursement for PA programs		Lack of parks, paths or pools in neighborhood		Number of barriers	
	*B*^a^	*p*	*B*	*p*	*B*	*p*	*B*	*p*	*B*	*p*	*B*	*p*	*B*	*p*	*B*	*p*
Age (per year)	** − .010**	**.001**	** − .023**	** < .001**	** − .012**	** < .001**	** − .011**	**.001**	** − .019**	** < .001**	** − .024**	** < .001**	− .003	.236	** − .048**	** < .001**
Sex^b^	− .208	.061	** − .258**	**.039**	− .078	.412	− .051	.666	− .140	.283	− .293	.037	− .032	.696	− .518	.084
BMI (per 1 kg/m^2^)	**.015**	**.010**	**.015**	**.023**	**.017**	** < .001**	.004	.515	.008	.215	**.017**	**.019**	**.010**	**.022**	**.056**	** < .001**
Educational level^c^	** − .113**	**.046**	** − .163**	**.010**	** − .133**	**.006**	** − .187**	**.002**	** − .148**	**.026**	** − .232**	** < .001**	− .032	.445	** − .481**	**.002**
Current work status^d^	** − .136**	**.046**	** − .331**	** < .001**	** − .149**	**.010**	** − .213**	**.003**	** − .238**	**.003**	** − .205**	**.017**	− .041	.413	** − .650**	** < .001**
Cancer type																
Colorectal^e^	.153	.154	− .023	.842	.171	.056	.036	.745	.128	.298	.057	.670	− .024	.758	.334	.239
Prostate^f^	.253	.058	.086	.564	**.229**	**.044**	.135	.342	.260	.097	.222	.187	− .031	.747	.634	.078
Time since diagnosis (per month)	− .005	.171	.000	.935	− .004	.228	− .005	.207	− .006	.191	.000	.929	− .003	.393	− .013	.230
Current treatment^g^	.087	.339	.125	.220	.053	.501	.115	.231	.160	.134	.219	.056	.038	.572	.462	.059
Chemotherapy^h^	− .029	.658	.071	.324	.021	.701	.062	.363	.044	.564	− .046	.575	.008	.868	− .036	.835
Radiotherapy^h^	.038	.547	.043	.550	**.147**	**.007**	**.193**	**.005**	.123	.102	.155	.054	.075	.111	**.465**	**.007**
Hormone therapy^h^	− .052	.582	− .067	.532	.032	.695	− .077	.446	− .107	.337	− .151	.206	− .017	.810	− .341	.217
Co-morbidities^i^	.074	.202	**.189**	**.004**	.087	.081	**.137**	**.028**	**.155**	**.024**	**.156**	**.033**	.046	.279	**.381**	**.015**
5A score for PA counseling^j^	** − .075**	**.009**	− .041	.210	.005	.838	** − .075**	**.015**	** − .117**	**.001**	− .015	.682	.002	.925	− .126	.106

^a^Regression coefficients are displayed as unstandardized beta coefficients

^b^0: female; 1: male

^c^0: no degree or (lower-) secondary education degree; 1: diploma qualifying for university or university degree

^d^0: homemaker, retired, on sick-leave or unemployed; 1: currently working or student

^e^Cancer type, dummy-coded; 0: breast cancer, prostate cancer; 1: colorectal cancer

^f^Cancer type, dummy-coded; 0: breast cancer, colorectal cancer; 1: prostate cancer

^g^0: currently not receiving chemo, radio, or hormone therapy; 1: currently receiving chemo, radio, or hormone therapy

^h^0: never having received this treatment; 1: having received or currently receiving this treatment

^i^0: no co-morbidities; 1: ≥ 1 co-morbidities

^j^Weighted sumscore for PA counseling based on 5A framework; higher values indicate more comprehensive counseling

### Association of Structural Barriers and Insufficient Post-diagnosis PA

Results of the logistic regression analyses examining the association of perceived structural barriers and insufficient post-diagnosis PA are displayed in Table 4. The analyses revealed that individuals who perceived structural barriers as more impeding for their PA were significantly more likely to be insufficiently active post-diagnosis, above and beyond the effect of established PA determinants. Among all structural barriers, “lack of information material” was most strongly related to insufficient post-diagnosis PA with each increase in level of perceived impediment increasing the likelihood of not meeting PA guidelines post-diagnosis by 38% (CI 1.15; 1.65, *p* < 0.001). Higher perceived impediments by other structural barriers, except “lack of parks, paths or pools in the neighborhood,” increased the likelihood of not meeting PA guidelines post-diagnosis by 16–30% (all *p’s* < 0.05). A higher number of perceived barriers was also significantly associated with insufficient post-diagnosis PA with each additional barrier increasing the likelihood of not meeting PA guidelines post-diagnosis by 14% (CI 1.07; 1.21, *p* < 0.001).

**Table 4:** Linear regression results on socio-demographic and medical determinants of perceiving structural barriers

		Model for barrier: lack of information material			Model for barrier: lack of PA offers for people with cancer			Model for barrier: lack of PA offers overall			Model for barrier: lack of possibility for medical clearance				
		*OR*	CI	*p*	*OR*	CI	*p*	*OR*	CI	*p*	*OR*	CI			*p*
Structural barrier^a^	Per 1 step	**1.38**	**(1.15; 1.65)**	**<.001**	**1.21**	**(1.04; 1.42)**	**.016**	**1.27**	**(1.02; 1.58)**	**.032**	**1.22**	**(1.03; 1.43)**			**.019**
Age	Per 1 year	1.00	(0.98; 1.01)	.779	1.00	(0.98; 1.01)	.805	1.00	(0.98; 1.01)	.657	1.00	(0.98; 1.01)			.675
Sex	Female	Reference			Reference			Reference			Reference				
	Male	0.73	(0.40; 1.33)	.303	0.74	(0.41; 1.34)	.323	0.73	(0.40; 1.32)	.296	0.71	(0.39; 1.30)			.267
BMI	Per 1 kg/m²	1.06	(1.02; 1.09)	.001	1.06	(1.02; 1.09)	.001	1.05	(1.02; 1.09)	.003	1.06	(1.02; 1.09)			.001
Educational level^b^	Lower	Reference			Reference			Reference			Reference				
	Higher	0.67	(0.49; 0.91)	.009	0.67	(0.50; 0.91)	.010	0.68	(0.51; 0.93)	.014	0.68	(0.50; 0.92)			.012
Cancer type	Breast	Reference			Reference			Reference			Reference				
	Colorectal	2.66	(1.59; 4.45)	<.001	2.74	(1.64; 4.55)	<.001	2.55	(1.53; 4.27)	<.001	2.70	(1.62; 4.51)			<.001
	Prostate	2.90	(1.44; 5.83)	.003	3.09	(1.55; 6.18)	.001	2.79	(1.39; 5.62)	.004	2.99	(1.49; 6.00)			.002
Time since diagnosis	Per 1 month	0.97	(0.95; 0.98)	<.001	0.96	(0.94; 0.98)	<.001	0.96	(0.94; 0.98)	<.001	0.96	(0.94; 0.98)			<.001
Co-morbidities	None	Reference			Reference			Reference			Reference				
	≥1	1.44	(1.05; 1.96)	.023	1.40	(1.03; 1.92)	.034	1.41	(1.03; 1.92)	.033	1.44	(1.05; 1.97)			.022
Pre-diagnosis MVPA	0–149 min/week	Reference			Reference			Reference			Reference				
	≥150 min/week	6.50	(4.73; 8.93)	<.001	6.08	(4.43; 8.34)	<.001	6.05	(4.41; 8.31)	<.001	6.21	(4.53; 8.52)			<.001
5A score^c^	Per 1 step	0.72	(0.61; 0.85)	<.001	0.70	(0.60; 0.82)	<.001	0.70	(0.60; 0.83)	<.001	0.70	(0.60; 0.83)			<.001

		Model for barrier: Lack of expert contact person			Model for barrier: Lack of reimbursement for PA programs			Model for barrier: Lack of parks, paths or pools in neighborhood			Model for number of barriers				
		*OR*	CI	*p*	*OR*	CI	*p*	*OR*	CI	*p*	*OR*		CI	*p*	
Structural barrier^a^	Per 1 step	**1.30**	**(1.12; 1.51)**	**.001**	**1.16**	**(1.00; 1.33)**	**.043**	1.21	(0.95; 1.55)	.119	**1.14**		**(1.07; 1.21)**	**<.001**	
Age	Per 1 year	1.00	(0.98; 1.03)	.821	1.00	(0.98; 1.01)	.907	1.00	(0.98; 1.01)	.591	1.00		(0.99; 1.02)	.987	
Sex	Female	Reference			Reference			Reference			Reference				
	Male	0.67	(0.37; 1.23)	.194	0.68	(0.38; 1.25)	.214	0.70	(0.39; 1.26)	.237	0.75		(0.41; 1.36)	.341	
BMI	Per 1kg/m²	1.06	(1.02; 1.09)	.001	1.06	(1.02; 1.09)	.001	1.06	(1.02; 1.09)	.001	1.05		(1.02; 1.08)	.004	
Educational level^b^	Lower	Reference			Reference			Reference			Reference				
	Higher	0.66	(0.49; 0.89)	.007	0.68	(0.51; 0.93)	0.14	0.66	(0.49; 0.90)	.008	0.68		(0.50; 0.92)	.013	
Cancer type	Breast	Reference			Reference			Reference			Reference				
	Colorectal	2.79	(1.66; 4.70)	<.001	2.82	(1.69; 4.72)	<.001	2.64	(1.59; 4.39)	<.001	2.63		(1.58; 4.38)	<.001	
	Prostate	3.21	(1.58; 6.50)	.001	3.08	(1.54; 6.18)	.002	2.94	(1.47; 5.86)	.002	2.88		(1.44; 5.77)	.003	
Time since diagnosis	Per 1 month	0.97	(0.95; 0.99)	.001	0.96	(0.94; 0.98)	<.001	0.96	(0.94; 0.98)	<.001	0.96		(0.95; 0.98)	<.001	
Co-morbidities	None	Reference			Reference			Reference			Reference				
	≥1	1.44	(1.05; 1.97)	.023	1.41	(1.03; 1.92)	.033	1.45	(1.06; 1.98)	.020	1.36		(1.00; 1.86)	.053	
Pre-diagnosis MVPA	0-149 min/week	Reference			Reference			Reference			Reference				
	≥150 min/week	6.37	(4.63; 8.78)	<.001	6.21	(4.53; 8.53)	<.001	6.19	(4.52; 8.50)	<.001	6.38		(4.66; 8.75)	<.001	
5A score^c^	Per 1 step	0.72	(0.61; 0.85)	<.001	0.69	(0.59; 0.82)	<.001	0.71	(0.60; 0.83)	<.001	0.70		(0.60; 0.83)	<.001	

*OR* odds ratio, *CI* 95% confidence interval, *PA* physical activity, *MVPA* moderate-to-vigorous physical activity

Separate regression models for each structural barrier. Dependent variable “insufficient post-diagnosis PA”, i.e., not meeting PA guidelines of 150 min MVPA per week. Bold values indicate *p* < .05

^a^Structural barrier as indicated in column heading; impediment for PA measured on a scale from 0 “not at all” to 3 “very strongly”; higher values indicate higher perceived impediment for PA

^b^*Lower*: no degree or (lower-) secondary education degree; *Higher:* diploma qualifying for university or university degree

^c^Weighted sumscore for PA counseling based on 5A framework, higher values indicate more comprehensive counseling

### Association of Structural Barriers and Change Patterns for Insufficient Post-diagnosis PA

Investigating the association of structural barriers and insufficient post-diagnosis PA in subgroups defined by whether or not individuals were meeting PA guidelines pre-diagnosis however revealed that the association differed between the two change patterns for insufficient post-diagnosis PA (Table 5). The analyses among participants who were meeting PA guidelines before the diagnosis yielded that those perceiving higher impediment by structural barriers were significantly more likely to become insufficiently active: All except for two barriers increased the likelihood of not meeting PA guidelines post-diagnosis by 20–39% with each increase in level of impediment (all *p’s* < 0.05). Looking at the number of barriers, each additional perceived barrier was associated with a 16% increase in the likelihood of becoming insufficiently active (CI 1.07; 1.26, *p* < 0.001). In contrast, none of the structural barriers were significantly associated with the pattern of remaining insufficiently active among previously insufficiently active individuals (all *p’s* > 0.05).

**Table 5:** Logistic regression results on impact of structural barriers on insufficient post-diagnosis physical activity (PA), separately for both possible PA change patterns

	**Becoming insufficiently active**^b^			**Remaining insufficiently active**^c^		
Lack of^a^	*OR*	CI	*p*	*OR*	CI	*p*
Information material	**1.37**	**(1.01; 1.71)**	**.005**	1.34	(0.98; 1.84)	.068
PA offers for people with cancer	**1.22**	**(1.00; 1.49)**	**.048**	1.21	(0.93; 1.56)	.157
PA offers overall	**1.32**	**(1.00; 1.74)**	**.047**	1.18	(0.83; 1.69)	.359
Possibility for medical clearance	1.14	(0.96; 1.45)	.108	1.30	(0.98; 1.73)	.071
Expert contact person	**1.39**	**(1.16; 1.67)**	** < .001**	1.14	(0.87; 1.49)	.336
Reimbursement for PA programs	**1.20**	**(1.01; 1.43)**	**.044**	1.10	(0.86; 1.40)	.449
Parks, paths or pools in neighborhood	1.35	(0.97; 1.89)	.078	1.19	(0.83; 1.70)	.338
Number of barriers	**1.16**	**(1.07; 1.26)**	** < .001**	1.10	(0.97; 1.24)	.137

*OR* odds ratio, *CI* 95% confidence interval, *PA* physical activity

Separate regression models with dependent variable “insufficient post-diagnosis PA,” i.e., not meeting PA guidelines of 150 min moderate-to-vigorous PA (MVPA) per week, for each structural barrier. All models adjusted for age, sex, BMI, educational level, cancer type, time since diagnosis, co-morbidities, and 5A score for PA counseling. Bold values indicate *p* < .05

^a^Impediment for PA by structural barriers measured on a scale from 0 “not at all” to 3 “very strongly”; higher values indicate higher perceived impediment for PA

^b^Subgroup analysis for participants meeting PA guidelines pre-diagnosis (≥ 150 min MVPA/week)

^c^Subgroup analysis for participants not meeting PA guidelines pre-diagnosis (< 150 min MVPA/week)

## Discussion

In the present study, we investigated the perception of structural barriers for PA among people with breast, prostate, or colorectal cancer by identifying determinants and examining the association of structural barriers and insufficient post-diagnosis PA in terms of different PA change patterns. Overall, 30–60% of participants indicated to perceive impediment for their PA by structural barriers, which was particularly salient among individuals who were younger, currently not working, had a lower educational level, a higher BMI, co-morbidities, and reported less comprehensive exercise counseling by physicians. With regard to post-diagnosis PA, we found that individuals who perceived higher impediments by structural barriers were more likely to be insufficiently active post-diagnosis. However, the association did not apply to participants who remained insufficiently active pre- and post-diagnosis but only to those who were meeting PA guidelines pre-diagnosis and became insufficiently active thereafter.

Only a few studies have investigated the role of structural barriers for PA among people with cancer so far, thereby mainly focusing on environmental factors [13, 16, 17]. Consistent with these studies, minority of individuals perceived a lack of overall PA offers or exercise opportunities in the neighborhood as a barrier to PA in our study. However, the results of our study suggest that beyond this, individuals with cancer seem to perceive structural barriers that relate to a lack of disease-adjusted PA offers and support as much more impeding for their PA with about half of participants indicating that a lack of therapeutic programs reimbursed by health care insurances, a missing contact person who is specialized in exercise oncology counseling and treatment as well as a lack of PA offers specifically designed for people with cancer kept them from performing PA. The latter is in line with findings from a previous study where 52% of participants reported to be unaware of available PA programs [18]. The prevalence of this kind of structural barriers points out the desire for more guidance, support, and possibilities to exercise in a setting that is specifically tailored to the individual’s health condition. As previous research regarding facilitators for PA among people with cancer has also yielded preferences for PA programs that are tailored according to individual abilities and disease states [24–26], the uptake and adherence to PA after a cancer diagnosis might be enhanced by the development and implementation of individually adjusted PA programs.

We further investigated which individuals, based on their socio-demographic and medical characteristics, perceived structural barriers as particularly impeding for their PA. The analyses yielded a stronger impediment among people with a higher BMI and lower educational levels, which is in line with previous research [15]. As these individuals tend to be less inclined to exercise in general [8, 27, 28], structural barriers could already influence their intention to initiate PA. In accordance with Romero et al. [15], impediment by structural barriers was also higher among younger individuals. Of note, a younger age has previously been shown to be associated with higher post-diagnosis PA levels [9, 29, 30]. A possible explanation for the nevertheless stronger perception of structural barriers could be that daily obligations like work or family duties keep younger individuals from searching for possibilities to overcome perceived barriers. With regard to medical determinants, we found that participants with co-morbidities reported higher levels of impediment by structural barriers. This extends previous literature where co-morbidities as such were identified as barriers for PA among people with cancer [12, 13]. Physical restrictions and health issues could thus not only pose a challenge to exercise but also lead to more insecurity about the safety of PA, which points out the need for particular guidance on how and where to safely perform PA in consideration of individual health conditions.

As expected, the perception of structural barriers was strongly associated with insufficient post-diagnosis PA, which supports the results of a study among people with colorectal cancer [17]. However, while Lynch et al. rather focused on suitable PA facilities and the local environment and calculated an overall score for structural barriers [17], we further specified the contribution of thematically different structural barriers. The results indicated that a lack of information material was most strongly related to insufficient post-diagnosis PA. Providing information material appears as a convenient and time-efficient PA intervention method and the relevance of exercise counseling by HCP as patients’ preferred source of information has been widely acknowledged in the literature [19, 21, 31, 32]. However, given that physicians often do not feel qualified or lack the time to discuss PA [33–35] and additional information material has been revealed as a crucial component of effective exercise counseling [36], it appears necessary to establish and enhance the access to high-quality self-educational material for interested individuals. Our results further indicated that not the local environment but rather a lack of PA offers specifically designed for people with cancer, expert contact persons, and financial reimbursement kept individuals from performing PA. These findings have important practical implications as they do not only highlight the need for suitable exercise programs for people with cancer but also emphasize the relevance of informative communication as well as medical and financial support in this regard.

Considering that previous PA behavior has been shown to be the strongest predictor of post-diagnosis PA [8], we further explored the role of structural barriers as a function of different change patterns for insufficient post-diagnosis PA. Interestingly, the analyses showed that the perception of structural barriers was not significant for the pattern of remaining insufficiently active among individuals who were not meeting PA guidelines pre-diagnosis. On the one hand, this could be explained by a floor effect; i.e., individuals with no pre-diagnosis PA did not face any structural barriers due to their inactivity. On the other hand, other factors such as low intentions and low perceived behavioral control may be stronger predictors for post-diagnosis PA behavior among individuals with low pre-diagnosis PA levels. In contrast, structural barriers seemed to pose a greater challenge for the maintenance of sufficient PA levels in the presence of disease-related issues among previously active individuals. This could explain why individuals who were highly active before the diagnosis tend to show stronger decreases in their PA behavior from pre- to post-diagnosis than those with lower pre-diagnosis PA levels [37, 38]. Thus, it appears important to acknowledge that particularly previously active individuals, who are usually more likely to continue pursuing PA after the diagnosis, seem to be impeded in their PA by structural barriers and hence in need of support to overcome these barriers.

### Strengths and Limitations

To our knowledge, this is the first study to comprehensively investigate determinants and the impact of structural barriers on post-diagnosis PA in people with cancer and the results reveal important new insights. Nevertheless, some limitations have to be considered. Our study comprised a large sample, but is missing detailed data on socioeconomic status of participants and lacking variance in terms of receipt of surgery, both factors that have been shown to be relevant for PA behavior among people with cancer and could be meaningful predictors of the perception of structural barriers [15, 39]. Data on PA was collected as retrospective self-report and could therefore be biased. Further, the assessment of PA barriers might not be extensive. While participants were asked to rate the perception of seven different structural barriers, other potential barriers to PA like health-related, social, or personal factors have not been analyzed in terms of impediment for PA. Therefore, it is not possible to compare the contribution of structural barriers to other barrier themes, which would be an interesting research question for future studies. Further, it would be interesting for future research to investigate how structural barriers interact with psychological variables like intentions or attitudes towards PA in their effect on post-diagnosis PA. Finally, the cross-sectional design of our study does not allow causal inferences.

## Conclusion and Implications

In conclusion, our study emphasizes the contribution of different structural barriers to insufficient post-diagnosis PA among people with breast, prostate, and colorectal cancer. The analyses revealed differences in the perception of structural barriers with regard to individuals’ socio-demographic and medical characteristics, which are relevant for a more specific targeting of PA interventions. The associations of almost all structural barriers with insufficient post-diagnosis PA, particularly among individuals who were sufficiently active before the diagnosis, do not only highlight the need for the implementation of individually adjusted PA programs but further point out the importance of improvements in patient education as well as medical guidance and financial support with regard to PA after a cancer diagnosis.

## Footnotes

1 Note that no differentiation was made between aerobic and resistance exercises.

## Declarations

### Research Involving Human and Animal Participants

All procedures performed in studies involving human participants were in accordance with the ethical standards of the institutional and/or national research committee and with the 1964 Helsinki declaration and its later amendments or comparable ethical standards.
